# Prevalence of Autism Spectrum Disorder (ASD) in Inpatient Adolescent Psychiatric Population

**DOI:** 10.1007/s10803-023-05923-w

**Published:** 2023-04-06

**Authors:** Graciela Kriegel, Sayani Paul, Kate H. Leonard, Paul Sandor

**Affiliations:** 1 Ontario Shores Centre for Mental Health Sciences, 700 Gordon St, L1N5S9 Whitby, ON Canada; 2 Youthdale Treatment Center and Youthdale Research Institute, Toronto, Canada

**Keywords:** Autism spectrum disorder, Prevalence, Comorbidity, Psychiatric, Adolescents

## Abstract

The prevalence of autism spectrum disorder (ASD) has increased in recent decades, much of which is related to changes in diagnostic criteria, and greater awareness among professionals and parents. Using a prospective cross-sectional study design, this study explores the prevalence of ASD among 173 adolescents admitted to two psychiatric facilities in Canada, and its association with some early pre and perinatal risk factors. The overall prevalence of ASD in the psychiatric population was 11.56% compared to 1.52% in children and youth in Canada. While prenatal and perinatal factors were not significantly associated with ASD, we found a frequent association of ASD with different comorbid psychiatric conditions. These findings further our knowledge in planning and management of ASD among this population.

## Introduction

Autism Spectrum Disorders (ASD) are a set of complex neurodevelopmental disorders that affect social, communication and behavioural development as well as other associated areas such as sensory processing. Even though ASD is a lifelong condition, it is often difficult to detect early; with mean age at diagnosis being 38 ± 15 months (Valicenti-McDermott et al., 2012) and a higher occurrence among males than females, at about a 3:1 ratio (Loomes et al., 2017). Although the etiology of ASD is considered to be largely genetically determined yet a variety of prenatal and perinatal factors such as advanced maternal and paternal age, maternal place of birth, low birth weight, short gestation period (Gardener et al., 2011; Sandin et al., 2016) may be associated with the risk of ASD.

The epidemiology of ASD is changing fast. In the past two decades, there has been a clear increase in reported prevalence of ASD. Based on 2018 data, there were approximately one in 44 children diagnosed with ASD in the United States, with ASD being four times more prevalent among boys than amongst girls (Maenner et al., 2021). Similarly, in Canada, approximately one in 66 (1.52%) children and youth were diagnosed with ASD (Ofner et al., 2018). Some factors contributing to the continuous rise in the prevalence of ASD include: case definition (i.e. changes in the conceptualization of autism), diagnostic substitution to access more comprehensive services through the change in diagnosis, better methods of case identification, increased public awareness, more services and policies (Saracino et al., 2010). This certainly raises concerns regarding the accuracy of the epidemiological description of ASD that affect the planning for appropriate services for this population (Baxter et al., 2015).

ASD classification under the DSM-5 shifted from the triadic to the dyadic symptom grouping and merging some categories such as autistic disorder, Asperger’s disorder and pervasive developmental disorder-not otherwise specified (PDD-NOS) into a single diagnostic category (American Psychiatric Association, 2013). This led to change in reported prevalence of ASD that now subsumes all three groups under one label. On the other hand a number of individuals diagnosed with ASD according to DSM-IV-TR may no longer qualify for diagnoses under the new DSM-5 criteria (Smith et al., 2015). In a systematic review, Smith et al., (2015) found between 50 and 75% of individuals would maintain ASD diagnosis as per the DSM-5 and that individuals with intelligence quotient (IQ) of 70 or more and/or those with diagnosis of PDD-NOS or Asperger’s Disorder would not qualify under the new diagnostic criteria resulting in a decrease in overall prevalence of ASD. A prospective study demonstrated excellent specificity and good sensitivity relative to DSM-IV criteria, strongest for autistic disorder but poor for those that had met criteria for Asperger Disorder and PDD-NOS (Mazurek et al., 2017).

### Psychiatric Comorbidities Associated with ASD

Adolescents with ASD frequently present varied emotional, behavioural or cognitive disturbances along with the symptoms that commonly define autism. Research confirms the frequent co-occurrence of ASD with at least one psychiatric diagnosis (Weiss et al., 2018), with a rate between 70% (Simonoff et al., 2008) to 83% (Joshi et al., 2010). The most common psychiatric diagnoses associated with ASD include Attention Deficit Hyperactivity Disorder (ADHD), Oppositional Defiant Disorder (ODD), anxiety and mood disorders (Brookman-Frazee et al., 2017).

### ASD and Inpatient Hospitalizations

ASD is associated with significant mental health care utilization and subsequent healthcare expenditures (Ames et al., 2020; Righi et al., 2018) with almost 11% requiring psychiatric hospitalizations (Wink et al., 2018). Young adults (18–24 years) have frequent visits to outpatient primary care (Ames et al., 2020), paediatrician, family physician, psychiatrists and emergency department (ED) compared to their peers (Weiss et al., 2018); particularly, they may have four times more frequent ED visits compared to those without ASD (Liu et al., 2017). Reasons for inpatient hospitalization could be due to environmental causes (e.g. lack or disruption of treatment, adjustment disorders), organic causes (e.g. seizures, painful medical conditions) (Guinchat et al., 2015), psychiatric causes (i.e. catatonia, major depressive disorder, bipolar disorder, schizophrenia and/or schizoaffective disorders, emotional dysregulation, aggression) (Conner et al., 2021; Guinchat et al., 2015; Schlenz et al., 2015; Siegel & Gabriels, 2014) as well as injuries ranging from self inflected injury to falls and accidents, wounds and cuts and poisoning/ingesting foreign objects (Schlenz et al., 2015).

Limited research on psychiatric hospital treatment models for youth with ASD indicates that psychotropic drugs (Ames et al., 2020; Wink et al., 2018), ADHD medication and sleep aids are the most frequently used treatment modality (Wink et al., 2018). When treated in specialized inpatient psychiatric units compared to general units, adolescents with ASD have shown better outcomes in terms of reduction in behavioural problems, rehospitalization rates, ED and crisis service utilization two months post discharge (Taylor et al., 2019). This research suggests the need for specialized treatment for this population. Some of the predictors associated with inpatient hospitalizations among youth with ASD include aggression towards others and psychotropic polypharmacy (Modi et al., 2015), low adaptive functioning, higher severity of social-affective ASD symptomatology, having a non-married/non-domestic partnered primary caregiver, presence of mood disorder diagnoses and sleep problems (Righi et al., 2018) and preadolescent and adolescent age (Schlenz et al., 2015).

There is limited research on adolescents with ASD receiving care in inpatient psychiatric settings. Further, detecting the psychiatric comorbidities among individuals with ASD is particularly important to ensure they receive the most appropriate interventions and services. To this end, this study aims to identify the prevalence of – (1) ASD in children presenting to psychiatric facilities, (2) early pre- and peri-natal risk factors associated with ASD, and (3) comorbid psychiatric conditions associated with ASD. The findings may inform service providers and families to anticipate the services and resources that families will require during these developmental stages for young adults with ASD. The following hypotheses were tested – (1) the prevalence of ASD in an inpatient child and adolescent psychiatric population will be significantly higher than that reported in the general population and (2) pre and peri-natal factors such as gestational age, low birth weight and advancing parental age are more likely to be associated with ASD in the inpatient population.

## Methods

### Participants and Setting

This study was conducted at two inpatient psychiatric settings in Ontario, Canada – Ontario Shores Centre for Mental Health Sciences (Ontario Shores) and Youthdale Treatment Centre. Ontario Shores is a specialized mental health hospital offering a range of mental health treatment and services to individuals, including adolescents whose lives are impacted by a mental illness. Youthdale Treatment Centre is a community agency providing inpatient treatment to children, youth and their families struggling with complex mental health needs. Data were collected from 173 adolescents between the ages of 12 to 18 years who had an IQ higher than 70 and were admitted to the inpatient units at one of the two study sites. Participants were included in the study independently of having an ASD diagnosis. Parents or caregivers of the study participants were included in the study as key informants. The study received ethics approval from the research ethics boards (REB) at respective study sites.

### Study Design

We used a prospective, cross sectional study design to meet the study objectives.

### Measures

We used the Childhood Autism Rating Scale (CARS) and Krug Asperger’s Disorder Index (KADI) to detect and diagnose autism disorder and Asperger’s disorder in addition to an in-depth clinical evaluation. Both measures are well validated and have strong psycho-metric properties (Chlebowski et al., 2010; Krug & Arick, 2003). The demographic information included participants’ medical, developmental and behavioural history, as well as information from parents - their age at the time of participants’ birth, pre and perinatal information and mothers’ countries of birth.

### Data Collection

All patients who met the eligibility criteria were invited to participate in the study. Data were collected by clinicians at respective study sites using the same eligibility and diagnostic criteria based on DSM-IV-TR and DSM 5 and same procedures. A participant was diagnosed to have ASD based on comprehensive clinical assessment that included: (a) detailed developmental history obtained from their parents and/or guardians, (b) observation on the unit and (c) results on the KADI and/or the CARS. All diagnostic and symptom ratings were reviewed and confirmed by a licensed child and adolescent psychiatrist (GK). Based on these different components of clinical assessments, a patient was diagnosed with ASD.

- under DSM IV TR - when they met a total of six (or more) items from (1), (2), and (3), with at least two from (1), and one each from (2) and (3) of the DSM IV TR criteria (American Psychiatric Association, 2000) and
- under DSM 5 - when they had persistent deficits in each of three areas of social communication and interaction (A.1. through A.3. plus at least two of four types of restricted, repetitive behaviors (B.1. through B.4. of the DSM 5 criteria (American Psychiatric Association, 2013). All participants from Ontario Shores were diagnosed using DSM 5, while DSM IV TR and DSM 5 were used to diagnose participants at Youthdale Treatment Centre.

Comorbid diagnosis was obtained from discharge report made by the treating psychiatrist. In this study, low birth weight was defined as less than 2000 g (Pinto-Martin et al., 2011) and the advanced maternal and paternal age was defined as 35 years or older (Ben Itzchak et al., 2011). In total, 105 participants from Youthdale Treatment Centre and 68 participants from Ontario Shores were included.

### Data Analyses

Data collected at Youthdale Treatment Centre were transferred to Ontario Shores for analysis purposes. Chi-square and *t*-tests were conducted to compare the participants across two study sites. In order to assess the psychiatric comorbidities associated with ASD among our study participants, the study sample of two study sites were analyzed separately. Data were managed and analysed using IBM SPSS Statistics 23.

## Results

### Prevalence Rate of ASD

Twenty (eight from Ontario Shores and 12 from Youthdale Treatment Centre) of 173 participants received a diagnosis of ASD, i.e. the overall prevalence rate of ASD in our study sample was 11.56% (*X*^*2*^ = 9.309, p = 0.002). This is significantly higher than the most recent prevalence rate of ASD (1.52%) in the general Canadian population (1 in 66) (Ofner et al., 2018). The prevalence of ASD in our study sample at both sites were quite similar − 11.7% and 11.4% respectively.

### Age

**Fig. 1:**
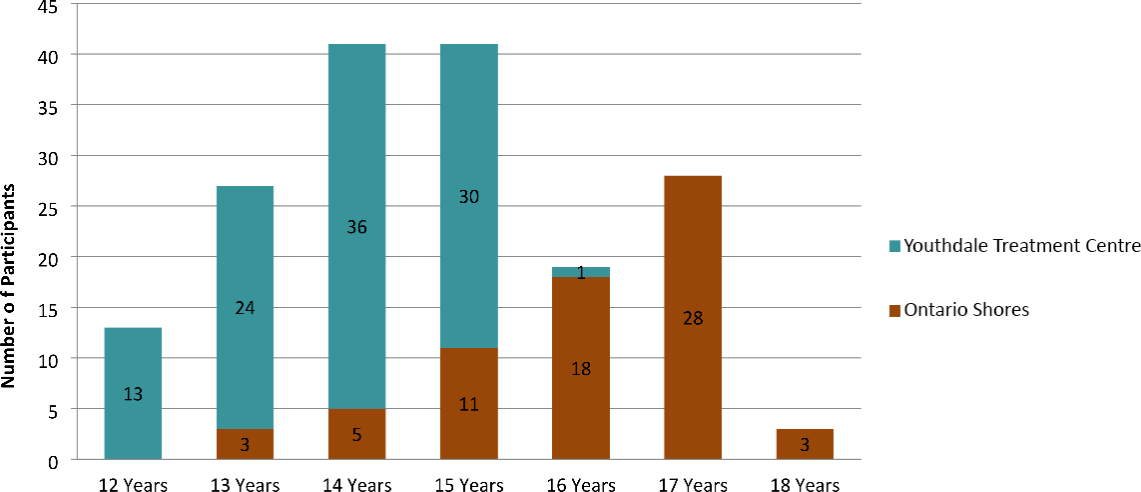
Age breakdown of study participants across two sites

There was a statistically significant difference (*t *= 13.04, p = 0.001) in participants’ age (see Fig. 1). The mean age of participants with ASD at Ontario Shores was 16.06 years (SD = 1.2) and at Youthdale Treatment Centre 13.83 years (SD = 1.01). The combined mean age of participants with ASD at both study sites was 15.11 years (SD = 1.5).

### Sex

More males (13 of 20 participants with ASD) met the diagnostic criteria for ASD and this difference was statistically significant *X*^*2*^ = 4.821, p = 0.028. Notably, the sex ratio was rather different in ASD group as compared to group without ASD. Boys predominated in ASD group 65% (n = 13) while in non-ASD group boys represented only 39% (n = 60). This result is presented in aggregate form because the sample at each site was too small.

### Prenatal and Perinatal Factors

We found no statistically significant differences in the gestational ages of participants with ASD and those without ASD (*X*^*2*^ = 0.209, p = 0.901). Although there appeared to be a trend among participants with ASD to have low birth weight, the difference compared to non-ASD participants failed to reach statistical significance (p = 0.06). We found that the maternal (*X*^*2*^ = 0.198, p = 0.906) or paternal age (*X*^*2*^ = 0.976, p = 0.614) at the time of participant’s birth, in our ASD sample was not advanced compared with non-ASD sample. When compared between two study sites, there were no significant differences with regards to gestational age (t=-1.719, p = 0.088), birth weight (t=-1.433, p = 0.154) and maternal (t = 0.640, p = 0.523) and/or paternal age (t=-0.777, p = 0.438) (See Table 1).

**Table 1:** Pre and Peri natal factors associated with participants with ASD.

Variable			Overall	With ASD	Without ASD
**Gestational Age (weeks)**					
n			149	16	133
Mean			38.53	37.94	38.60
Standard Deviation			3.38	3.02	3.43
**Birth Weight (kg)**					
n			144	15	129
Mean			3.22	2.89	3.26
Standard Deviation			0.95	0.87	0.96
**Maternal Age**					
n			159	17	142
Mean			28.23	28.00	28.26
Standard Deviation			6.22	5.31	6.35
**Paternal Age**					
n			151	16	135
Mean			31.80	32.53	31.71
Standard Deviation			7.30	6.02	7.46

**Fig. 2:**
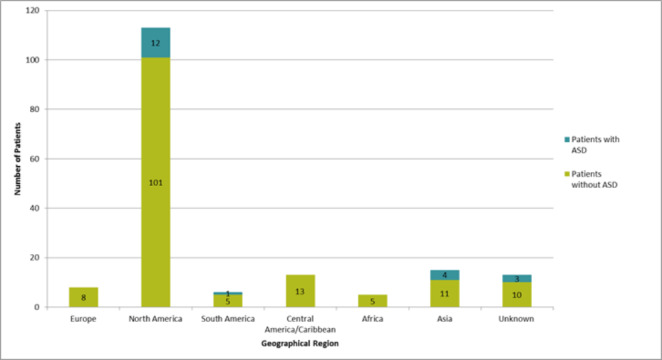
Distribution of maternal place of birth for participants with and without ASD.

For participants with ASD, maternal place of birth was not statistically associated with ASD diagnosis (*X*^*2*^ = 0.781, p = 0.377). Eight participants with ASD, reported their mother’s place of birth was outside North America or Europe (See Fig. 2), while 12 participants’ mother’s place of birth was in North America.

### Psychiatric Comorbidities

**Fig. 3:**
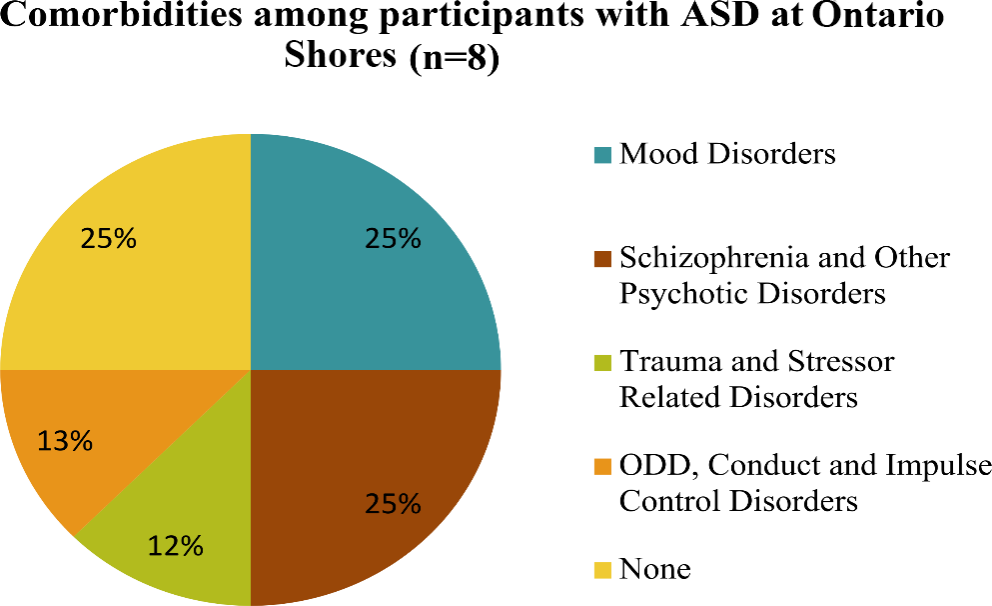
Psychiatric diagnoses of participants at Ontario Shores

Almost all participants at both sites were admitted due to their psychiatric comorbidities and/or behavioural problems, including risk of self harm and/or harm to others, although ASD was strongly implicated in the ability to communicate with the participants, thereby adding a layer of severity to the symptoms presented. At Ontario Shores, six out of eight (75%) participants with ASD had comorbid diagnosis. Two (25%) participants with ASD had a comorbid mood disorder and one (13%) had a comorbid diagnosis of ODD, while two participants were diagnosed with schizophrenia (25%), one (12%) with trauma related stress disorders and two (25%) had none (See Fig. 3).

**Fig. 4:**
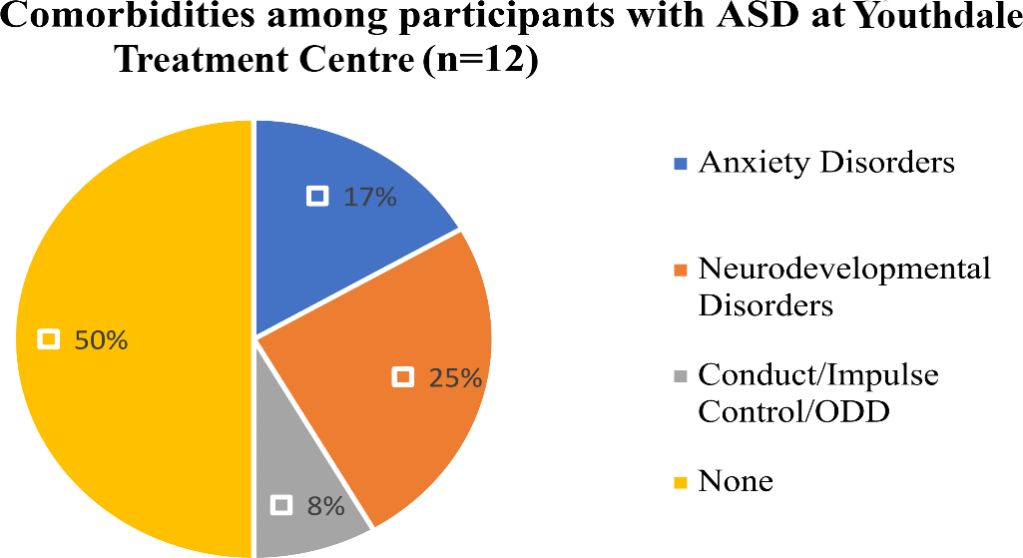
Psychiatric diagnoses of participants at Youthdale Treatment Centre

**Fig. 5:**
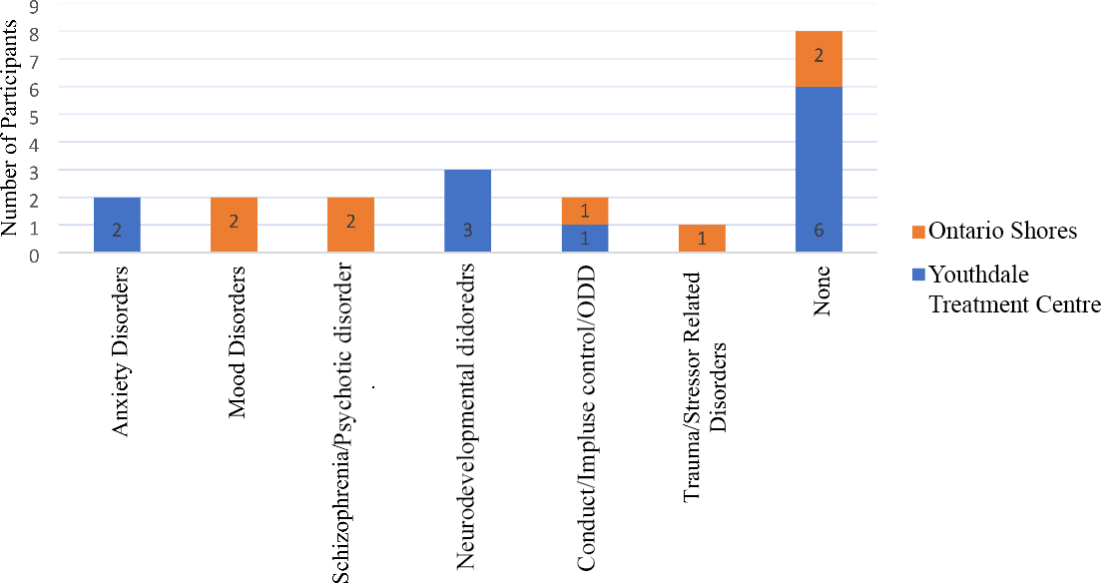
Comparison on psychiatric diagnoses among participants with ASD between Youthdale Treatment Centre and Ontario Shores

At Youthdale Treatment Centre, six (50%) participants with ASD had comorbid diagnosis. Two participants (17%) had anxiety disorders, three (25%) had neurodevelopmental disorders, one (8%) had conduct/impulse control/ODD. Six participants with ASD (50%) had no comorbid condition (See Fig. 4). Results showed participants with ASD at Ontario Shores had a greater number of comorbidities as compared to participants with ASD at Youthdale Treatment Centre (75% vs. 50%) (See Fig. 5).

**Fig. 6:**
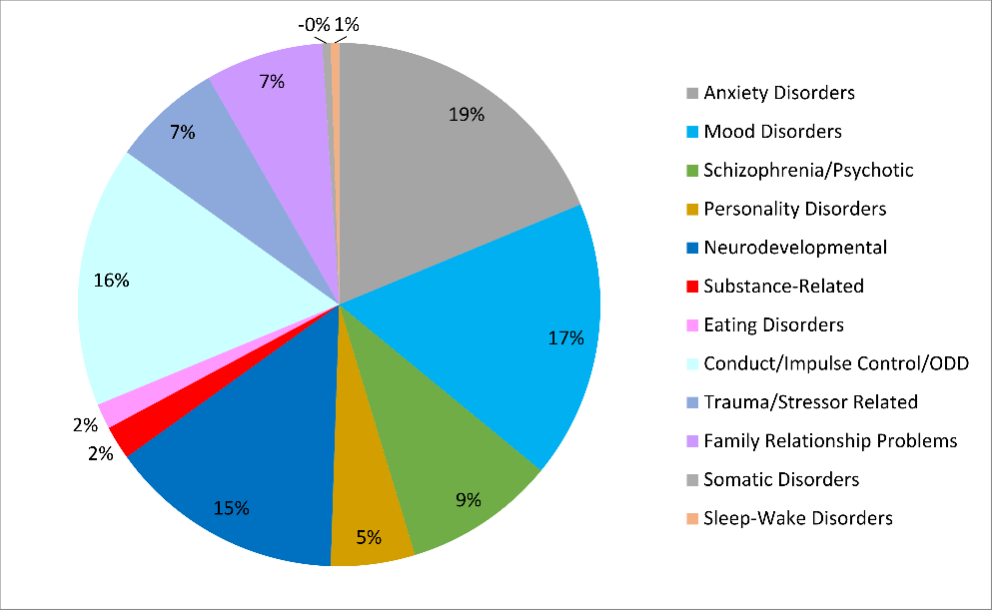
**Psychiatric Diagnoses of Participants without ASD**

Among those participants who did not have diagnosis of ASD, a total of 145 participants had different psychiatric diagnoses (See Fig. 6).

## Discussion

Our findings support the first hypothesis of higher prevalence of ASD (11.56%) in an inpatient psychiatric population compared to the general population (1.52%). Contrary to earlier studies (Sandin et al., 2016), we did not find a significant association between pre- and perinatal factors. There was a trend for participants with ASD to have lower birth weight but this failed to reach statistical significance, likely due to our relatively small sample size. Consistent with existing literature (Loomes et al., 2017; Taylor et al., 2019), we found participants with ASD were predominantly male. Literature, indicates that males are more often diagnosed with ASD at a ratio of 3:1 (Loomes et al., 2017) The difference in age between the two study sites could be attributed to the fact that the two institutions have different age criteria for admission; Youthdale Treatment Centre supports children and youth between 6 and 16 years, while Ontario Shores caters to children and adolescents between 12 and 18 years.

Participants with ASD from Ontario Shores were more likely to have at least one psychiatric comorbidity, compared to participants with ASD from Youthdale Treatment Centre (75% versus 50%), and the types of comorbidities varied at each site including diagnosis of mood and anxiety disorders, ODD, schizophrenia and stress related disorders, and neurodevelopmental disorders. This finding is somewhat consistent with existing research (Brookman-Frazee et al., 2017; Joshi et al., 2010; Ozbaran et al., 2021; Righi et al., 2018), although it is surprising that none of our participants had a diagnosis of ADHD in our study cohort. It is likely that patients with comorbid diagnoses are more likely to have behavioural problems that may have led to the need for inpatient treatment.

Since all participants were admitted due to their psychiatric and behavioural problems, behavioural modification therapies, psychopharmacotherapy and psychoeducation to families were used to address participants’ behavioural and psychiatric concerns. As a practice implication, the need to include ASD Care Pathway (ASD-CP) for future training of staff particularly in general psychiatric units, who may have to care of youth with ASD and comorbid psychiatric conditions is emphasized. ASD-CP pathway is known to reduce length of stay (LoS) (Kuriakose et al., 2018) and use of crisis interventions among patients with ASD (Cervantes, 2019; Kuriakose et al., 2018). The need to comparatively examine the efficacy of ASD-CP pathway in specialized psychiatric setting and general unit is warranted.

Findings highlight a higher prevalence of ASD in the inpatient population compared to the general population. In addition, the elevated prevalence of psychiatric comorbidity in the inpatient population, underscores the need to prioritize the availability, education and training of clinicians with expertise in the diagnosis and treatment of ASD and its comorbidities in inpatient settings, of both acute care and as tertiary psychiatric care centres. Aligning with the most recent guidelines for ASD recently published by the Lancet Commissions (Lord et al., 2022) we emphasize the need for stepped care model to identify and treat the comorbid conditions of ASD.

### Limitations

It is unknown whether our observed prevalence was influenced by the changes in diagnostic criteria, as some data were collected before DSM V was published in 2013. Second, not all patients with ASD consented to participate in the study, hence the prevalence we report may be an underestimate. We also did not include patients with IQ less than 70, potentially further reducing the prevalence rate we detected. Participants who were not accompanied by their immediate caregivers often failed to provide adequate information that were crucial for clinical assessments, thereby impacting the ASD diagnosis and subsequent prevalence rate. Another limitation of the study involves the failure to consider the phenomenon of ‘camouflaging’ i.e. masking autistic symptoms (Green et al., 2019). Girls with ASD are more likely to camouflage than boys (Green et al., 2019; Schuck et al., 2019) thereby potentially contributing to the difference in prevalence of ASD. Although, in this study all potential participants, both males and females were systematically assessed to diagnose ASD using CARS and KADI and clinical assessments, yet the possibility of girls camouflaging their autistic symptoms cannot be completely ruled out, that may impact our findings. To help clinicians in the management of hospitalized ASD patients, future studies examining their LoS and factors impacting the LoS is also recommended.

### Future Directions

In future, we recommend meta-analysis of existing interventional studies of psychiatric conditions in ASD patients to answer questions such as does the presence of ASD influence the outcome of psychosocial and pharmacological treatment of these psychiatric conditions? Is the outcome affected by the severity of ASD? Do the ASD patients respond in the usual manner to treatment? We also suggest a prospective randomised trial among inpatient population with and without ASD diagnosis with a focus on one of the most commonly associated psychiatric condition such as ADHD, anxiety or major depression (Joshi et al., 2010). Another area of research may explore the psychopharmacology aspect of ASD. Given that there is great interindividual variability in clinical response and adverse effect is observed in the ASD population (Persico et al., 2021), future research can further examine the psychopharmacological treatment of psychiatric comorbidities associated with ASD. Recognising the need for pertinent information around ASD treatment in psychiatric hospitals, future study with robust sample may focus on describing the characteristics of young adults diagnosed with ASD, role of pre and perinatal factors associated with ASD and factors contributing to their admission, as well as pharmacological and non-pharmacological management of the conditions during inpatient hospitalization. Additionally, with the increase in prevalence rate of ASD, another area of research would be to explore whether hospitalization rates of children and adolescents with ASD have also increased. Such study would have implication in ASD management and service delivery in the hospital as well as in the community.
